# Approaches to murine T cell isolation and activation

**DOI:** 10.1016/j.mocell.2025.100286

**Published:** 2025-10-14

**Authors:** Sangjun Lim, Sohyun Kum, Jin Ouk Choi, Joonbeom Bae, Soo Seok Hwang

**Affiliations:** 1 School of Biological Sciences, Seoul National University, Seoul 08826, Republic of Korea; 2 Institute of Molecular Biology and Genetics, Seoul National University, Seoul 08826, Republic of Korea; 3 Department of Biotechnology, College of Life Sciences and Biotechnology, Korea University, Seoul 02841, Republic of Korea

**Keywords:** Basic immunology, Fluorescence-activated cell sorting, Magnetic-associated cell separation, Murine T cell, T cell activation

## Abstract

In this MiniResource, we outlined practical approaches for preparing murine T cells, from isolation to in vitro activation, with emphasis on reproducibility and viability. While specific experimental conditions should be tailored to individual assays, the principles summarized here provide a framework for establishing robust T cell preparation across diverse research settings. By integrating standard methodologies with troubleshooting insights, this resource aims to support both basic immunological studies and future applications in T cell engineering.

## INTRODUCTION

**Fig. 1:**
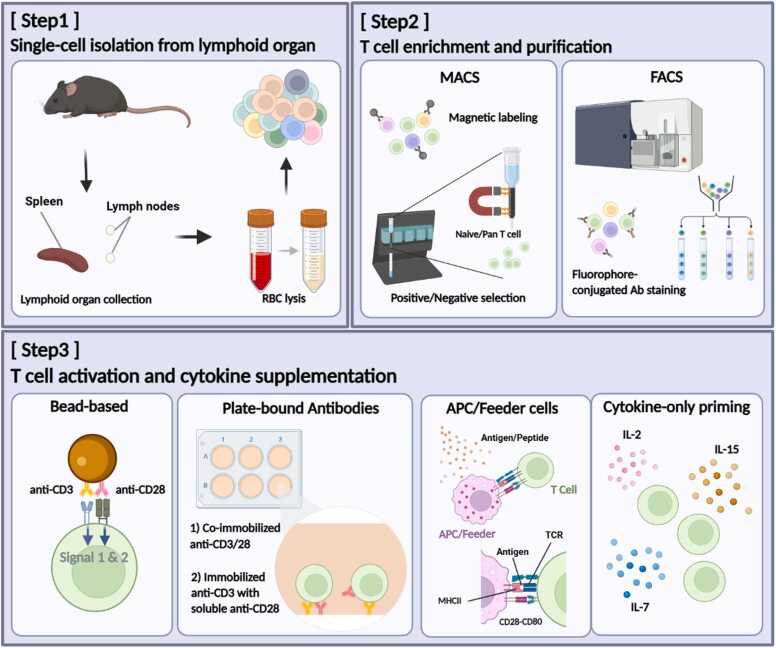
Schematic overview of murine T cell isolation/enrichment and in vitro activation. (Step1) Lymphoid organs are harvested, mechanically dissociated to generate single-cell suspensions, with red blood cells (RBCs) lysed. (Step2) T cells are enriched and/or purified mostly by MACS (magnetic-activated cell sorting; positive or negative selection) or by FACS (fluorescence-activated cell sorting) following staining with fluorophore-conjugated antibodies. (Step3) T cell receptor (TCR) and costimulatory signals can be delivered using antibody-coated beads or plate-bound antibodies; alternatively, primary antigen-presenting cells (APCs) provide peptide-MHC (major histocompatibility complex) together with costimulatory ligands (CD80/CD86) to approximate physiological stimulation.

T cells are central players in adaptive immunity, governing host defense, immune regulation, and therapeutic responses. Reliable preparation of T cells in mouse models underpins both mechanistic research and translational immunology. Over the years, diverse methodological approaches have been developed to improve yield, purity, and reproducibility in T cell isolation and activation within mouse models. These include magnetic-associated cell separation (MACS) and fluorescence-activated cell sorting (FACS) of lymphoid organ-derived single-cell suspensions, followed by antibody- or antigen-presenting cell (APC)-based activation systems with cytokine supplementation (Miltenyi et al., 1990, Sutermaster and Darling, 2019, Trickett and Kwan, 2003; Fig. 1). In this MiniResource, we provide practical guidelines for isolating and activating murine T cells, particularly for researchers less familiar with primary T cell handling. This article also serves as the entry point of a 2-part series: here we focus on establishing a high-quality T cell population, while the subsequent article addresses viral transduction strategies for genetic engineering, such as transduction for chimeric antigen receptor-T cell (CAR-T) development (Ayala Ceja et al., 2024, Vormittag et al., 2018).

## METHODS FOR T CELL ISOLATION

Secondary lymphoid organs, such as spleens and lymph nodes, are standard sources for T cell preparation (Malhotra et al., 2013). To obtain single-cell suspension, tissues are gently dissociated on a 70-µm strainer and washed with cold phosphate-buffered saline, preferably with the addition of fetal bovine serum to enhance cell viability. Following cell collection by centrifugation, red blood cells are removed with ammonium-chloride-potassium lysing buffer to enrich lymphocyte populations and improve isolation efficiency. The resulting cells are subsequently resuspended and filtered once more through a nylon mesh to remove cellular aggregates.

MACS and FACS are among the most widely used approaches for T cell isolation (Table 1). MACS uses antibody-conjugated microbeads that target the cognate antigen expressed on the cell surface (Grutzkau and Radbruch, 2010, Khurana et al., 2022). Two principal strategies are employed for T cell isolation: positive and negative selection. In positive selection, T cells are directly labeled using lineage markers such as CD3, CD4, or CD8. Negative selection method targets other cell subsets, thereby depleting non-T cells or unintended T cell populations. Both strategies are easily accessible, showing moderately high yield and purity along with simple workflow. However, it should be noted that the positive selection method may interfere the activation state of T cells via cell-bound antibodies, and the residual beads cannot be avoided when directly performing imaging and flow cytometric analysis (Laghmouchi et al., 2020).

**Table 1:** Comparison of methods for murine T cell isolation

Method	Principles	Strength	Limitations	Yield/purity	Recommended use
MACS (positive selection)	Cells conjugated with specific antibodies-bead conjugates are strongly captured	High purity, simple workflow	Targeting CD3 or TCR can induce unintended activation; beads may affect downstream workflow	Variable yield; purity > 95%	Preclinical CAR studies, needing high purity of chimeric antigen receptor transduced T cells
MACS (negative selection)	Antibody-bead cocktails deplete non-T lineages, leaving unlabeled T cells “untouched”	Preserve native signaling state; minimal receptor engagement	Highly dependent on kit quality; costly	High yield; purity: 90%-95%	Functional assays (proliferation, differentiation, engineering), unbiased activation
FACS	Droplet-based high-purity physical sorting	Precise subset-specific sorting; removal of apoptotic and dead cells	Time-consuming; requires highly expensive FACS instrumentation; lower total yield	Variable yield; purity > 98%	Subset-specific mechanistic studies

CAR, chimeric antigen receptor; FACS, fluorescence-activated cell sorting; MACS, magnetic-associated cell separation.

FACS can significantly enhance the purity of T cell isolation (Schraivogel et al., 2022). As various fluorochrome-conjugated antibodies can be utilized for this method depending on the purpose, precise and subset-specific T cell isolation is available (Matsumoto et al., 2023). This is particularly useful for mechanistic studies of naïve, memory, or regulatory T cells. Nonetheless, relatively reduced yield and viability due to prolonged processing may affect downstream assays (Cossarizza et al., 2021, Yi et al., 2023). To address this issue, pre-enrichment with MACS before FACS can mitigate these drawbacks (Cossarizza et al., 2021).

Suboptimal experimental conditions can compromise cell viability during T cell isolation, subsequently impairing T cell integrity and functionality. To minimize this issue, all experimental steps should be carried out without delays, and cell suspensions must be carefully handled under controlled temperature (4°C) to avoid apoptosis. Processing steps that impose mechanical or chemical stress, such as tissue dissociation, require optimization to reduce cellular damage.

## APPROACHES TO T CELL ACTIVATION AND CYTOKINE SUPPLEMENTATION

T cell activation begins when TCR signaling exceeds the activation threshold upon recognition of peptide-MHC on APCs (Irvine et al., 2002, Zhu et al., 2010). This event drives naïve T cells from quiescence into clonal expansion and differentiation (Au-Yeung et al., 2014, Hwang et al., 2020). Various in vitro activation strategies provide a relatively homogeneous starting population for controlled functional studies and downstream applications such as CAR-T engineering (Zhang et al., 2017; Table 2).

**Table 2:** Approaches to T cell activation and cytokine supplementation

Method	Principles	Cytokine	Strength	Limitations	Notes
Bead-based (anti-CD3/CD28)	Beads immobilized with anti-CD3/CD28 enforce uniform polyclonal activation	IL-2±IL-7 or IL-15	Highly reproducible; scalable; simple hands-on workflow	Bead carry-over can interfere with imaging/flow; induce overactivation due to crosslinking	Adjust bead/cell ratio to intended activation strength
Plate-bound Abs	Anti-CD3 is adsorbed to tissue-culture plate; costimulation is added as soluble or immobilized anti-CD28	IL-2	Precise control over stimulus strength; inexpensive; no beads removal step needed	Relatively inconsistent activation strength due to well-to-well variability from coating heterogeneity	Coating density critical for cell viability and consistency
Antigen-presenting cells	Delivering physiologic multisignal activation and enabling antigen-specific expansion	IL-2, IL-15	Physiological multisignal stimulation; antigen-specific expansion	Variable; time-consuming due to isolation of APC and/or maturation/differentiation	More suitable for translational experiments; peptide pulsing process required
Cytokine-only priming	Maintains cells with resting state	IL-7, IL-15	Preserves stem-like state	Slow activation; lower yield due to non-expansion	Useful for Tscm or memory-like T cell preservation

APC, antigen-presenting cell; Tscm, T memory stem cells.

A simple and practical way to monitor activation is by examining cell morphology under a light microscope. Activated T cells undergo blastogenesis, becoming visibly larger than resting cells. This size increase is typically evident by ∼20 hours poststimulation, providing a convenient checkpoint before proceeding to downstream assays such as viral transduction or functional testing.

Antibody-based systems mimic the 2-signal requirement: TCR engagement via anti-CD3 (Signal 1) and costimulation via anti-CD28 (Signal 2) (Jenkins and Johnson, 1993). Plate-bound methods require immobilized anti-CD3 with soluble or immobilized anti-CD28, whereas bead-based systems provide more uniform and sustained activation, though at higher cost. Because activation strength strongly affects outcomes ranging from poor proliferation to functional exhaustion, empirical titration of stimulation dose is essential (Wang et al., 2008).

Alternatively, activation can be achieved using APCs, particularly dendritic cells (Jobin et al., 2025, Storni and Bachmann, 2003). This approach mimics physiological interactions and is suitable for translational studies. However, variability and the labor-intensive preparation of APCs—requiring isolation, differentiation, and antigen loading—limit routine use. Moreover, APC-derived cytokines and costimulatory ligands may confound experiments focused on T cell–intrinsic mechanisms (Gutcher and Becher, 2007).

Cytokine supplementation (Signal 3) is indispensable for sustained activation and survival (Mescher et al., 2006). IL-2 provides essential survival and proliferation signals through STAT5-dependent pathways (Ross and Cantrell, 2018, Villarino et al., 2022). For prolonged culture, IL-7 and IL-15 improve viability and promote memory-like characteristics (Choi et al., 2022, Pilipow et al., 2015, Tan et al., 2001). When maintenance of a quiescent state is required, IL-7 and IL-15 in the absence of TCR stimulation can support cell viability while preserving a resting phenotype (Choi et al., 2025, Sprent and Surh, 2011).

## CONCLUDING REMARKS

We outlined core methodologies for murine T cell isolation and activation, emphasizing reproducibility and viability across experimental contexts. While individual conditions require optimization, the principles highlighted here offer a practical foundation for downstream applications such as functional assays and T cell engineering. For issues related to yield and viability, troubleshooting strategies are available for consultation (Table 3). Read in tandem with the following MiniResource on viral transduction, these protocols together provide an integrated pipeline from T cell preparation to genetic modification.

**Table 3:** Troubleshooting guide for T cell isolation and activation

Problem	Possible causes	Solution
Low T cell viability after single-cell isolation	Mechanical and chemical stress during single-cell suspension	• Tissue must be grinded more gently • Add ice-cold PBS with serum as needed during tissue grinding to reduce friction-induced heat • Adjust ACK lysis duration/buffer volume
Low postsort yield during FACS	Excessively high event rate of sample acquisition during sorting procedure	• Adjust the flow rate to minimize cell loss • Pre-enrich the sample with MACS using less antibodies/beads
Reduced T cell viability after in vitro TCR stimulation	Strong TCR signaling may cause activation-induced cell death	• Optimize appropriate dose for anti-CD3/28
Cell death after prolonged culture	Depletion of nutrients in the culture medium Exhaustion of T cells due to long exposure of TCR stimulation	• Adjust cell density before T cell stimulation • Change or add media • Adjust proper days of culture and once T cells are activated, maintain them in anti-CD3/CD28-free medium to prevent exhaustion (supply suitable cytokines)

ACK, ammonium-chloride-potassium; FACS, fluorescence-activated cell sorting; MACS, magnetic-associated cell separation; PBS, phosphate-buffered saline.

## Author Contributions

**Sangjun Lim:** Writing – review & editing, Writing – original draft. **Jin Ouk Choi:** Writing – review & editing, Funding acquisition. **Sohyun Kum:** Writing – original draft, Visualization. **Soo Seok Hwang:** Writing – review & editing, Writing – original draft, Supervision, Project administration, Funding acquisition, Conceptualization. **Joonbeom Bae:** Writing – review & editing, Funding acquisition.

## Declaration of Competing Interests

The authors declare that they have no known competing financial interests or personal relationships that could have appeared to influence the work reported in this paper.
